# Laboratory test ordering in inpatient hospitals: a systematic review on the effects and features of clinical decision support systems

**DOI:** 10.1186/s12911-020-01384-8

**Published:** 2021-01-18

**Authors:** Sahar Zare, Zahra Meidani, Mohammad Shirdeli, Ehsan Nabovati

**Affiliations:** 1 Health Information Management Research Center (HIMRC), 5th of Qotb -e Ravandi Blvd, Kashan University of Medical Science, 87159-73449 Kashan, Iran; 2 Department of Health Information Management and Technology, Kashan University of Medical Sciences, Kashan, Iran; 3 Health Information Management, Human Resources Development Research Center, Shiraz University of Medical Sciences, Shiraz, Iran

**Keywords:** Clinical decision support system, Test ordering, Hospitals, Laboratories, Inpatients

## Abstract

**Background:**

Studies have revealed inappropriate laboratory testing as a source of waste. This review aimed at evaluating the effects and features of CDSSs on physicians' appropriate laboratory test ordering in inpatient hospitals.

**Method:**

Medline through PubMed, SCOPUS, Web of Science, and Cochrane were queried without any time period restriction. Studies using CDSSs as an intervention to improve laboratory test ordering as the primary aim were included. The study populations in the included studies were laboratory tests, physicians ordering laboratory tests, or the patients for whom laboratory tests were ordered. The included papers were evaluated for their outcomes related to the effect of CDSSs which were categorized based on the outcomes related to tests, physician, and patients. The primary outcome measures were the number and cost of the ordered laboratory tests. The instrument from The National Heart Lung and Blood Institute (NIH) was used to assess the quality of the included studies. Moreover, we applied a checklist for assessing the quality and features of the CDSSs presented in the included studies. A narrative synthesis was used to describe and compare the designs and the results of included studies.

**Result:**

Sixteen studies met the inclusion criteria. Most studies were conducted based on a quasi-experimental design. The results showed improvement in laboratory test-related outcomes (e.g. proportion and cost of tests) and also physician-related outcomes (e.g. guideline adherence and orders cancellation). Patient-related outcomes (e.g. length of stay and mortality rate) were not well investigated in the included studies. In addition, the evidence about applying CDSS as a decision aid for interpreting laboratory results was rare.

**Conclusion:**

CDSSs increase appropriate test ordering in hospitals through eliminating redundant test orders and enhancing evidence-based practice. Appropriate testing and cost saving were both affected by the CDSSs. However, the evidence is limited about the effects of laboratory test CDSSs on patient-related outcomes.

## Background

The results of laboratory tests have an important impact on patients’ care, as they influence physicians’ decisions including admission, drug orders, and discharge as well as monitoring and managing the vast majority of diseases. However, studies indicate that diagnostic tests are being used inappropriately as a meta-analysis result showed that almost 20% of laboratory tests are over-utilized and 45% are under-utilized [1]. A study has indicated that only 1–5% of chemistry tests and 1–3% of hematology tests have led to an action; action in this study meant any alternation from what would have been done without the test result [2]. Moreover, about 70% of residents, in one study were reported that they were ordering unnecessary daily laboratory tests [3].

Inappropriate test ordering can increase the risk of false positive results as well as medical errors [4]. Overutilization can potentially cause patient discomfort including phlebotomy-induced anemia [5]. Underutilization can also result in delayed or missed diagnosis. Studies have found that a vast majority of claims both in outpatients and emergency department belongs to missed diagnosis resulting in death or serious harm to patients [6, 7]. Overcrowded diagnostic services, increased length of stay (LOS), and waste of valuable healthcare resources are amongst other consequences of inappropriate testing [8–10]. Conversely, it imposes a lot of costs to healthcare as 3% of health care expenditures in the USA belong to laboratory testing [11–13].

Information technology [IT] has provided some solutions to decrease inappropriate laboratory tests ordering. Some of these technologies are electronic medical record (EMR) [14], electronic health record (EHR) [15], computerized physician order entry (CPOE) [16], and clinical decision support systems (CDSS) [17]. Of all these, CDSS has more potential to support physicians when deciding about ordering a test or interpreting the results. However, studies have shown inconsistent results about the impact of CDSSs on physicians’ performance and patients outcomes [18, 19]. Thus, there is a need for a scoping review on the effects of CDSSs on ordering appropriate laboratory tests.

Studies evaluating the impact of CDSSs on diagnostic testing showed no improvement in clinical outcome but small positive improvement on physicians behavior regarding diagnostic test ordering [20, 21]. There are two similar systematic reviews focusing on laboratory test ordering specifically. The first is Maillet et al. study [22] which addressed the IT impact on laboratory tests ordering process in primary healthcare. This study did not focus on the effectiveness of CDSSs rather it focused on some specific IT interventions. It also included the studies conducted in primary healthcare. The second systematic review by Delvaux et al. [23] included the studies conducted in diverse healthcare settings (i.e. primary healthcare, hospital outpatient, and hospital inpatient). They found that CDSSs had little or no effect on clinical outcomes but some effects on physician compliance rate. Neither of the studies has investigated the features of the included CDSSs mentioned as a suggestion in Delvaux et al. study [23]. Taking into account all studies conducted in inpatient hospitals and aimed at improving laboratory testing process, without considering study designs, might produce different results. Furthermore, features of successful CDSSs need to be investigated. Thus, the goal of current study was to conduct a systematic review on the effects and features of CDSSs on physicians' appropriate laboratory tests ordering in inpatient hospitals.

## Method

### Research question

Do CCDSSs improve practitioners' appropriate laboratory test ordering in hospitals?

### Search strategy and study selection

A search strategy was developed using keywords, MeSH terms, and major subject headings to identify published papers in the literature and adaptations were made for each database. Four databases were queried: Medline (through PubMed), SCOPUS, Web of Science, and Cochrane. We considered studies published till 21 January 2020 without any time limitation. The search strategy consisted of a combination of keywords and Mesh terms related to clinical laboratory services (laboratory test utilization), CDSSs, and hospitals. The search strategy is presented as supplementary (Additional file 1: supplementary A).

After removing duplicates, two authors (SZ and MS), working independently, selected the papers based on eligibility criteria. Titles and abstracts were screened for inclusion. The full text of potentially relevant papers was obtained, and both inclusion and exclusion criteria were considered. The reference lists of the identified papers were also searched to include any other paper missed during the electronic searches. Authors resolved disagreements through discussion and consensus, and any remaining disagreements were resolved by another author (EN).

### Study selection criteria

#### Inclusion criteria

##### Type of studies

A variety of evaluation study designs were included: randomized controlled trials (RCTs)*,* non-randomized controlled clinical trials (CCTs), prospective observational studies, before-after*,* and interrupted time series (ITS).

##### Type of population

The study populations in the included studies were laboratory tests, physicians ordering laboratory tests, or the patients for whom laboratory tests were ordered.

##### Types of interventions

Studies using CDSSs as an intervention to improve laboratory test ordering as the primary aim were included. In current study, a CDSS is considered as a health information technology system designed to provide assistance to physicians at the time of decision-making. CDSSs can facilitate access to data which are required to make decisions, provide reminders while a patient encounters, assists in both recognizing a diagnosis and entering appropriate orders, and alerts healthcare providers when new patterns in patient data are observed [22, 24]. In studies with multifaceted interventions, the effects of CDSS intervention were considered independently and the cases where separating the CDSS impact was impossible were excluded.

##### Type of outcomes

The included papers were evaluated for their outcomes related to the effect of CDSSs, which were categorized based on test-related, physician-related, and patient-related outcomes. These outcomes include: diagnostic yield and diagnostic detection rate, the number and cost of laboratory test ordered, laboratory turnaround time (TAT), STAT tests, guideline adherence for laboratory test ordering, physicians knowledge and attitude toward laboratory testing, patients outcome (e.g. patients safety, readmissions, death, length of stay and disposition). Test-related outcomes were the proportion of tests, cost of tests, test intervals, number of STAT request, and laboratory TATs. Physician-related outcomes include diagnostic yield and diagnostic detection rate, adherence or order cancellation after the reminders (or overriding the reminders), and physicians knowledge and attitude. Patient-related outcomes were patients' complications, patients' disposition, length of stay (LOS), and mortality rate.

#### Exclusion criteria

Exclusion criteria were studies published in any languages rather than English, conducted in outpatient or primary care settings, used as interventions rather than CDSS, conducted in an unreal clinical environment or based on a scenario (in a simulated setting i.e. to test a system). Moreover, all retrospective studies were excluded.

### Quality assessment

The National Heart Lung and Blood Institute (NIH) quality assessment tools for each type of studies [25] were used to assess the methodological quality of the included studies. The variety of study designs necessitated the use of different NIH quality assessment tools, That is Quality Assessment of Controlled Intervention Studies, case–control studies, and before-after studies with no control group. NIH tool categorizes studies as good, fair, or poor. Included studies were independently assessed by two reviewers (SZ & MS) and any disagreement over scoring was resolved by consensus.

Quality and features of the CDSSs were assessed using a checklist derived from Goldzweig et al. study [26]. This checklist considers the design and the degree of reporting information about CDSS and implementation characteristics. The checklist consists of three domains: CDSS design, data entry source, and implementation source.

### Data extraction

A form was designed to extract data from each of the included studies. For each study the following data were extracted: study design, sample size, intervention description, and results. One author (SZ) extracted data which were subsequently reviewed and confirmed by another reviewer (EN).

### Data analysis

A narrative synthesis was used to describe and compare the designs and the results of included studies. We categorized studies based on different features of CDSSs, outcome category, and effects of CDSSs. The effect of interventions were reported based on statistically significant positive, positive without statistical argument, no effect (not statistically significant), negative without statistical argument, or statistically significant negative [27]. Meta-analysis was not performed due to the variety of outcomes and results.

## Results

### Study selection (Fig. 1)

**Fig. 1:**
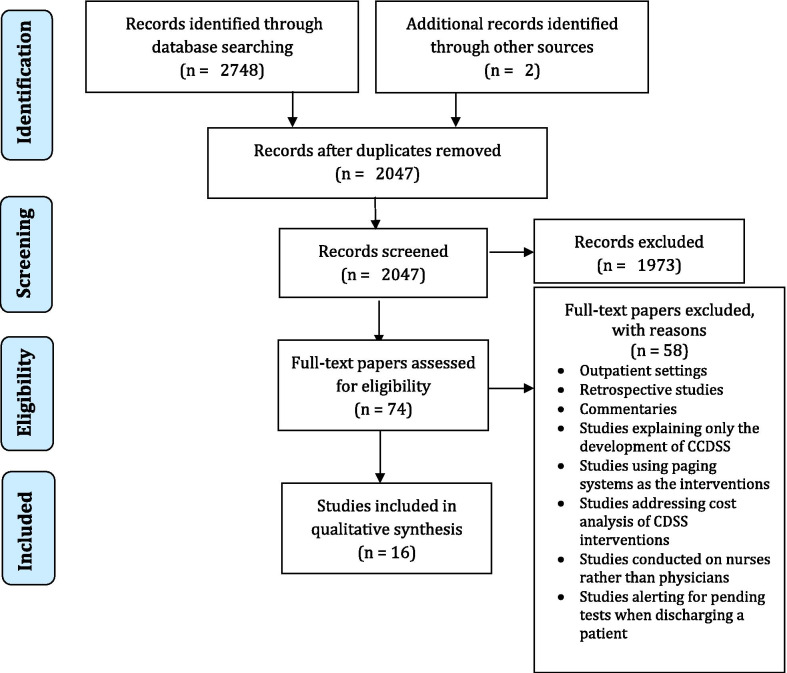
PRISMA flow diagram of the study selection

The literature search identified 2784 records, as well as two additional papers [28, 29] identified through other sources (snowball-search), 739 of which were duplicates. The papers were screened for eligibility by title and abstract, resulting in 74 potential papers for the full-text review. During the full-text reviewing, 58 papers were excluded. Finally, 16 studies were deemed eligible for inclusion.

### Characteristics of the included studies

A substantial number of the included studies were performed during the recent decade. Overall, 81.2% of the included studies were published after 2010 and, of these, 69.2% were published after 2015. Most of the included studies were conducted in the United States (n = 12, 75%); and one was conducted in each of the following countries: Canada [30], United Kingdom [31], Italy [32], and France [33] (Table 1).

**Table 1:** Characteristics of the included studies

References	Study design	Study duration	Setting	Population	Sample size	Intervention description	Main finding regarding the proportion of laboratory tests	Conclusion
Bates et al. [28]	RCT	4 months	A tertiary care hospital	Inpatients at the hospital	CG: 5886 patients IG:5700 patients	CPOE reminder: In the intervention group, if a test had previously been ordered within its test-specific interval, the physician received a reminder that the test had been performed recently or was pending; the result was showed if available. For the control group, duplication was determined in exactly the same way, but there was no reminder	(1) In the IG 69% cancelled the order after the reminder (2) In the CG, 51% of ordered redundant tests were performed, whereas in the IG only 27% of ordered redundant tests were performed (*P* < 0.001) (3) During the preceding period, 20.5% of target tests were performed earlier than specific intervals, whereas during the study period, this rate was significantly lower in the IG (18.5%, *P* = 0.004) but not in the control group (19.6%, *P* = 0.19) (4) In the 4-month period preceding the intervention, there were 4.84 target tests per admission, compared with 4.24 during the study period in the intervention group and 4.28 in the control group (both *P* < 0.0001)	Delivering reminders about orders for apparently redundant laboratory tests were effective. However, since many tests were conducted without corresponding computer orders and many orders were not screened for duplication overall effect was limited
Boon-Falleur et al. [31]	Before-after	6 months	A pediatric liver disease unit	Patients with liver transplant	Before: 42 patients After: 175 patients	A rule-based expert system allows static and dynamic requesting rules to be defined for different clinical classifications of patients. The static rules allow the definition of "baseline" proposals within a precise time schedule. Dynamic rules allow the system to react to results of previously ordered tests. The attending physician may accept or amend the system's proposals by adding or removing requests to the proposed schedule	(1) An increase of the total number of tests requested per patient was observed (2) An overall reduction in laboratory resources consumption for transplanted patients (27%) (3) A decrease in the percentage of "STAT" requested tests (− 44%) (4) The percentage of tests ordered in agreement with the protocols for those patients increased from 33% before the introduction of the expert system to 45% when the system was used	The clinicians’ perspective was that the system would increase the total benefits in clinical resources use, improve the management of laboratory data, and save time for doing laboratory ancillary tasks
Bridges et al. [34]	Before-after	6 months	A tertiary care hospital	Patient admitted to the department of medicine	Before: 674 patients After: 692 patients	The intervention consisted of displaying a computerized alert informing that the clinician is ordering a recently ordered test	(1) In the pre-intervention period, 53 (7.9%) were duplicated and post-intervention 18 (2.6%) were duplicated (*P* < 0.001) (2) The alert significantly reduce associated costs of duplicated acute hepatitis profile tests (*P* ≤ 0.001)	Computerized alerts may be effective in reducing redundant laboratory tests and enhancing efficiency of healthcare system
Dalal et al. [35]	Before-after	6 months	A teaching hospital	All TSH, T3, and T4 ordered in Department of Medicine	Before: 2611 tests After: 2454 tests	A clinical algorithm for CDS and Hard Stops were incorporated into the EMR to decline ordering freeT3 or freeT4 without an abnormal TSH, also certain exceptions were predefined. In addition, if the TSH was abnormal a reflex rule was triggered and could automatically order freeT3 and freeT4	(1) The fT3 to TSH ordering ratio similarly decreased by 55.2%, from 6.2 to 2.9% (*P* < 0.0001) (2) Post-intervention there was a decrease in the ratio of fT4 to TSH orders (fT4/TSH) of 35.2%, from 44.6% to 28.9% (*P* < 0.0001) (3) TFT/TSH pre-intervention ratio was 52.2%, which decreased by 39.1%, to 31.8% post-intervention (*P* < 0.0001)	By a clinical decision support about when to order TFTs, they observed a decrease in the number of unnecessary tests ordered
Eaton et al. [36]	Time-series	30 months	Hospital	Inpatient population admitted to general medicine service	Before: 14,193 patients After: 13,751 patients	Educational guide, nonintrusive ordering message, and noon conference. Appropriate indications for selected tests were incorporated into text accompanying the laboratory orders in hospital’s HER. Physicians could ignore the text and proceed with the order	(1) The rate of folate tests ordered per monthly admissions showed no significant level change at the time of the intervention with only a slight decrease in rate of 0.0109 (*P* = 0.07) (2) There was a 43% decrease in the rate of hepatitis C virus tests per monthly admissions immediately AI with a decrease of 0.0135 tests per monthly admissions (*P* = 0.02)	Nonintrusive CDSS do not have significant effect on utilization of laboratory test
Gottheil et al. [30]	Time series	12 months	A tertiary care hospital	Erythrocyte sedimentation rate orders	Not mentioned	Educational content and CDSS: a series of appropriateness criteria for Erythrocyte Sedimentation Rate was incorporated into CDSS	After CDS, ESR orders per week decreased from 386 to 151. When unlimited access was provided to select subspecialties, there was an increase in ESR orders per week to 241. This represents a decrease of almost 40% from baseline	Their quality improvement initiative could reduce inappropriate Erythrocyte Sedimentation Rate testing by computerized CDS
Klatte et al. [37]	Time series	12 months	A tertiary hospital, a 53-bed satellite facility	Specimens from children ≤ 12 months	485 specimens	Educational intervention, an evidence-based algorithm for appropriate clostridium difficile ordering, and CPOE requiring clinicians to mandatory complete 2 extra fields. Non diarrheal stool were automatically declined by laboratory, unless in cases with severe ileus or toxic megacolon	After the intervention, the average percentage of specimens tested dropped to 53.8%	Their CDSS intervention resulted in a sustained drop in the number of specimens tested, which saved laboratory and patient cost significantly. They observed no sustained change in clinicians’ ordering practices in spite of multiple educational efforts
Levick et al. [38]	Time series	6 months	Three not-for-profit hospitals	Patients with B-type natriuric peptide test	41,306 patients	CPOE with embedded CDS: The CDS intervention is an expert rule that searches the system for a B-Type natriuric peptide lab value for the patient. An advisory alert was indicated to the ordering clinician if there was a value for the test and it was within the current hospital stay	(1) The CDS intervention reduced B-Type natriuric peptide orders by 21% relative to the mean	Using CDSS alerts has the potential for improving care, but should be used judiciously and in the appropriate environment
Lippi et al. [32]	Before-after	6 months	A teaching hospital	A variety of tests requests including C reactive protein, TSH, ferritin, brain natriuretic peptide, etc	3539 test requests	CDSS: an electronic alert is automatically triggered by a potentially inappropriate test request. The alert contains a detailed explanation of the specific rule for appropriateness of the test	The total number of test requests violating the preset criteria of inappropriateness constantly decreased over time (26% in the first three months of implementation versus 17% in the following period; *P* < 0.001)	A CDSS alert may be effective to decrease the inappropriateness of laboratory test orders, generate significant cost saving and educate physicians to use laboratory resources more efficiently
Nicholson et al. [39]	Before-after non-equivalent control group	26 months	A tertiary-care pediatric hospital	Children < 36 months of age	Before: 141 patients After: 55 patients	An alert advising against ordering C. difficile tests in infants and young children based on the American Academy of Pediatrics recommendations. Physicians could override it optionally	(1) The average monthly testing rate significantly decreased for children 0–11 months old ( *P* < 0.001) and 12–35 months old (*P* < 0.001), but not for those children ≥ 36 months old (*P* = 0.3)	The average monthly testing rate for C. difficile for children < 35 months old decreased without complication after the use of a CPOE alert in those who tested positive for C. difficile
Niès et al. [33]	Time series	36 months	A university teaching hospital	Patients with hepatitis B antigen test	Before: 2888 patients After: 1572 patients	CDSS: The alert is triggered when one of the targeted serological tests for hepatitis B virus is selected to be ordered. The Serology-CDSS stores a record of its execution each time a physician selects a viral serology test order. An alert is displayed if the most recent result of the targeted laboratory test for the patient is less than 90 days old	(1) In pre-intervention period 15.5% of viral serology tests were unnecessarily repeated. During the intervention period, 15.8% were repeated. Before the intervention, the mean proportion of unnecessarily repeated HBs antigen tests increased by 0.4% per month (*P* < 0.001). After the intervention, a significant trend change occurred, with a monthly difference estimated at -0.4% (*P* = 0.02) resulting in a stable proportion of unnecessarily repeated HBs antigen tests	After CDSS implementation an immediately decrease was observed in the proportion of unnecessarily duplicate tests. CDSS alerts could also improve compliance rate
Quan et al. [40]	Before-after	24 months	An academic hospital	Patients with C. difficile infection test	Before: 284 tests After: 268 tests	Clinicians were required to verify the determined criteria for appropriate ordering of C. difficile infection test. A warning email was sent to the physicians ordering the test without appropriate approval	Baseline CDI testing rate declined from 284/10,000 to 268/10,000 patient-days post-intervention (*P* = 0.02). The intervention decreased inappropriate testing by 64%	The protocol increased appropriate testing as well as decreasing hospital-onset standardized infection ratio of C. difficile infection
Procop et al. [41]	Time series	24 months	The Cleveland Clinic	More than 1000 tests of all patients	Not mentioned	CDSS: This tool informs the provider that the test being ordered is a duplicate. It also block unnecessary duplicate test orders during the computerized physician order entry	(1) The proportions of reductions in the number of stool ova/parasite examinations was 54.1% (*P* < 0.0001) (2) The proportions of reductions in the number of Giardia/Cryptosporidium enzyme immunoassay tests was 22.58% (*P* = 0.2807) (3) The proportions of reductions in the number of stool culture tests was 49.1% (*P* < 0.0001)	Real-time interaction between the laboratory and the physician through CDS tools could decrease duplicate orders. It saves healthcare costs and should also increase patient satisfaction and well-being
Rosenbloom et al. [42]	Time series	5 years	An academic inpatient tertiary care facility	Clinicians at a university hospital	194,192 patients	The CDSS exhorted users to discontinue unnecessary tests recurring more than 72 h into the future (2) Education regarding appropriate indications for testing. (3) CDS and CPOE systems targeted only magnesium ordering, displayed recent results, limited testing to one instance per order, summarized indications for testing, and required users to select an indication	At baseline, there were 539 magnesium tests ordered per week. This decreased to 380 (*P* = 0.001) per week after the first intervention, increased to 491 per week (*P*, 0.001) after the second, and decreased to 276 per week (*P*, 0.001) after the third	A clinical decision support intervention intended to regulate testing increased test order rates as an unintended result of decision support
Rudolf et al. [43]	Time series	36 months	A tertiary care teaching hospital	Laboratory tests	61,644 laboratory test ordes	Alert in the CPOE system: the alert appeared in the CPOE each time an order with frequency greater than one occurrence was selected. The justification for the order was also captured by the CPOE, as providers were required to select one of three approved indications for the daily laboratory test or manually enter another indication	(1) 6,463 orders for recurrent daily laboratory tests were placed for a mean daily rate of 71.8 orders per day (2) AI 44,900 orders for recurrent daily laboratory tests were placed for a mean daily rate of 44.8 orders per day, representing a highly significant decrease in daily laboratory test ordering (3) Total inpatient test volumes were not affected	Our experience suggests auditing and continued feedback are additional crucial components to changing ordering behavior. Curtailing daily orders alone may not be a sufficient strategy to reduce in-laboratory costs
Samuelson et al. [44]	Before-after	16 months	Two academic medical hospitals	Patients evaluated for heparin-induced thrombocytopenia	Before: 265 patients After: 146 patients	CDSS: a decision-support tool required providers to calculate the 4Ts (heparin-induced thrombocytopenia risk) score prior to ordering laboratory-based tests for anti-PF4/heparin antibody enzyme-linked immunosorbent assay testing	(1) We observed a significant decrease from 43 tests/month before to 22 tests/month (* P* < 0.001) after the intervention (2) We observed a trend toward decrease in the proportion of tested patients with low 4Ts scores (66% vs 56%, *P* = 0.069),	Our study demonstrates that a clinical decision support tool embedded within the electronic ordering process can decrease unnecessary testing for heparin-induced thrombocytopenia

*CDSS* clinical decision support system, *CG* control group, *CPOE* computerized physician order entry, *ED* emergency department, *IG* intervention group, *RCT* randomized control trial, *TFT* thyroid function test, *TSH* thyroid stimulation hormone

### Quality assessment (Table 2)

**Table 2:** Quality assessment of the CDSSs

References	CDSS design				Data entry source		Implementation characteristic				
	Is it integrated with CPOE?	Does it give real time feedback at point of care?	Does the CDS suggest a recommended course of action?	CDSS Classification*	Is it automated through EHR?	Does clinical staff enter data specifically for intervention?	Was it pilot tested or used an iterative process of development/ implementation?	Was there any user training/clinician education?	Are the authors also the developers and part of the user group for the CDS?	Was there use of audit and- feedback (or other internal incentive)?	Are there any other implementation components not already discussed?
Bates et al. [28]	Yes	Yes	No	C	Yes	No	NM**	NM	Yes	No	50% of the tests with a computer order were not screened for redundancy because they were ordered as part of an order set
BoonFalleur et al. [31]	No	No	No	B	No	No	Yes	NM	NM	No	No
Bridges et al. [34]	Yes	Yes	No	B	NM	No	Yes	NM	No	No	Clinicians likely experienced an “adjustment” period once they became familiar with the alert,
Dalal et al. [35]	Yes	Yes	No	D	Yes	No	Yes	Yes	Yes	No	No
Eaton et al. [36]	Yes	Yes	No	B	No	NM	No	NM	NM	No	No
Gottheil et al. [30]	Yes	Yes	No	C	NM	No	Yes	NM	Yes	Yes	The importance of stakeholder engagement prior to the intervention and having decision leaders in each department to champion our cause
Klatte et al. [37]	Yes	Yes	No	D	Yes	Yes	Yes	NM	NM	No	No
Levick et al. [38]	Yes	Yes	No	B	No	No	NM	NM	Yes	No	Use of alerts should be used judiciously and in the appropriate environment
Lippi et al. [32]	Yes	Yes	No	B	NM	No	NM	NM	Yes	Yes	No
Nicholson et al. [39]	Yes	Yes	No	C	NM	Yes	NM	NM	Yes	No	No
Niès et al. [33]	Yes	Yes	No	C	Yes	No	Yes	NM	Yes	No	Testing options were constrained by unbundling serum metabolic panel tests into single components and reducing the ease of repeating targeted tests
Quan et al. [40]	Yes	Yes	No	D	NM	No	NM	NM	NM	Yes	No
Procop et al. [41]	Yes	Yes	No	D	NM	No	Yes	Yes	No	Yes	No
Rosenbloom et al. [42]	Yes	Yes	No	C	NM	Yes	NM	NM	Yes	Yes	Designers of CDS interventions should take into account the paradoxical prompting that such interventions might generate
Rudolf et al. [43]	Yes	Yes	No	C	NM	Yes	NM	NM	NM	Yes	Providers could use workarounds to place daily orders, entering the orders in a manner that would not trigger the audits. For example, placing staggered sets of orders to occur every other day or writing in daily orders on templates could have circumvented our auditing process and accounting of daily testing for this analysis
Samuelson et al. [44]	Yes	Yes	No	C	NM	Yes	NM	NM	NM	NM	No
**Sum**											
Yes No NM	15 1 0	15 1 0	0 16 0	A: 0 B: 5 C: 7 D: 4	4 3 9	5 10 1	8 1 7	2 0 14	8 2 6	6 9 1	

*Intervention Classification: “A” interventions provided information only; “B” interventions presented information on appropriateness or guidelines specifically tailored to the individual patient, often as a pop-up or alert. Some of these interventions also recommended alternative interventions, but did not include any barrier for the clinician to order the test; “C” interventions in general were similar to “B” interventions, but required the ordering clinician to justify with free text why they were overriding the decision support recommendation that a study was inappropriate (ie, a “soft stop”). “D” interventions included a “hard stop,” meaning the intervention prevented the clinician from ordering a test contrary to the CDS determination of inappropriateness, until additional discussion with or permission obtained from another clinician or pathologist

**Not Mentioned

One study was RCT [28], one case–control [39], and the others (n = 14) were quasi experimental studies (Additional file 2: supplementary B). Most of the included studies (n = 11, 68.7%) were of intermediate quality, the remaining were of good quality. The main limitations of the included studies were not being blinded (93.7% had not blinded assessors) and lack of a clear specified description of inclusion and exclusion criteria (43.7%). The results are presented as a supplementary (Additional file 2: supplementary B).

The quality assessments of the CDSSs are presented in Table 2. Almost all CDSSs were integrated with CPOEs (93.7%), providing real-time feedback (93.7%) without any recommended action (100%). Most CDSS classifications of the studies (43.7%) are in C category which required the ordering clinician to justify why they were overriding the provided decision support recommendation (see Table 2 legend). Four studies (25%) were integrated with and automated through EHR. Eight studies (50%) reported that they had tested CDS before implementation. Only two studies (12.5%) reported user training about the intervention; in other cases users were mostly trained about the indications required for ordering a specific test or similar things. Other characteristics, barriers, and facilitators affecting implementation of CDSS were: the role of order sets, “adjustment” period, stakeholder and champion leaders engagement, appropriate environment, ease of repeating targeted tests, testing options constrains, paradoxical prompting generated by CDSS, and daily orders which would not trigger the audits.

CDSS interventions were mostly in the form of a reminder about duplicate tests in a specific timeframe, rule-bases providing knowledge about when it is appropriate to order the specified test, or predefined appropriateness criteria physicians had to determine before ordering the tests. These interventions support physicians’ informed decision-making in the first step of testing process when they are deciding about ordering a test.

### Effects of CDSSs on outcomes (Table 3)

**Table 3:** Effects of CDSS interventions on laboratory testing outcomes

Outcome		Positive		No effect	Negative	
Category	Subcategory	Statistically significant	Demonstrated		Statistically significant	Demonstrated
Test-related	Proportion of tests	28, 34, 35, 36, 32, 39, 33, 40, 42*, 44	31, 36, 30, 37, 38, 41	31, 36, 43	42*	
	Cost of tests	34	28, 30, 37, 38, 32, 41			
	Test intervals	28				
	Number of STAT request		31			
Physician-related	Guideline adherence		31, 33			
	Orders cancellation after the reminders		28			
Patient-related	Patient complication			28, 39, 41		
	Patient disposition	34				
	LOS			34		
	Mortality rate	34				

*This study used three different CDSS intervention; two of which had positive impact and one of which had negative impact

The included studies had mostly investigated laboratory test-related outcomes. Generally, CDSS interventions showed positive effects on all outcomes.

### Laboratory test-related outcomes

All the included studies have investigated the effects of CDSSs on proportion of laboratory tests. In general, studies showed positive impact on proportion of laboratory tests. The reported proportion of reduction varied from 21% [38] to 55% [37] among the studies. The study by Boon-Falleur et al. [31], assessed as fair quality, applied a rule-based expert system for classified patients (Pre-transplant assessment, post-transplant assessment, and transplant monitoring) in liver transplant unit. The rule-based system increased laboratory utilization in pre-transplant assessment patients. The authors believed that, after the introduction of the system, physicians were asked to answer some precise questions, at patient admission, and it caused more often ordering of specialized diagnostic tests. However, it caused an overall reduction in laboratory resources consumption for transplanted patients. Eaton et al. [36] performed a multifaceted intervention in their good quality study indicating no effect on the rate of folate tests orders, but 43% reduction in the rate of hepatitis C virus tests. The study by Rudolf et al. [43] demonstrated that although recurrent daily laboratory tests reduced, the total tests volume remained unchanged. They stated that daily tests account for a small number of total tests; moreover, physicians may not decrease overall testing but instead shift testing to patients or conditions where it was more needed [43]. Rosenbloom et al. [42] used three CDSS interventions, two of which had a positive impact and one of which had a negative impact on magnesium ordering.

Cost of tests is also reported in half of the included studies. Results revealed that CDSSs had positive impact on reducing cost of tests. In most studies, except the one by Bridges et al. [34], with good quality, the reduction in the cost of laboratory tests was not analyzed with a statistical method [28, 30, 32, 37, 38, 41]. Test interval was only investigated in Bates et al., a good quality study [28], which showed a positive impact. "STAT" request of laboratory tests has only been investigated in a study by Boon-Falleur et al. [31] showing a positive impact.

### Physician-related outcomes

Three studies reported the outcomes related to guideline adherence and all indicated positive impacts of CDSS. Compliance rate was measured based on the proportion of cancelled orders after the provision of the reminders or recommendations by CDSS. Boon-Falleur et al. [31] showed that 78% of the total performed laboratory tests were proposed by the static assessment protocols. However, overall compliance to the dynamic protocols was 45%. Actually the compliance to the static rules was more in comparison with the dynamic rules. Bates et al. [28] showed that 69% of the proportion of laboratory orders was canceled after the provision of alert. They also found that only 27% of ordered redundant tests were performed. In the study by Nies et al. [33] the compliance rate to the displayed alerts was 24%. No outcome is reported regarding diagnostic detection rate and physicians’ knowledge.

### Patient-related outcomes

Patient-related outcomes were addressed in five studies. Cancellation of redundant tests based on the displayed alerts in some studies [28, 39, 41] resulted in little or no loss of clinical information as well as no complication. Bridges et al. [34] showed that patients with duplicate tests had higher mortality rate than those without duplicate tests. They also had a worse disposition after discharge, indicating that those with redundant tests were generally sicker. Redundant tests are those which are performed before a defined time frame (interval) for repeating that test [28, 34, 41]. Duplicate tests are also defined as a test that is ordered after a previous test of the same type that is unlikely to change clinical plan [34]. In this study, the patients LOS also remained unchanged after the intervention.

## Discussion

Generally, the studies were mostly of moderate methodological quality with only one RCT out of the 16 included studies as well as most studies being conducted after 2015. The majority of included studies were addressing the effect of CDSSs on laboratory test-related outcomes. The results showed improvement in laboratory test-related and physician-related outcomes. Patient-related outcomes were not well investigated in the included studies.

Most studies conducted after 2015 suggested a new research agenda in health information technology. It also indicates that attentions to resource utilization for appropriate usage of laboratory tests have been increased recently. It might also be attributed to limited resources as well as increased cost of healthcare. Healthcare resource utilization and the costs by different diseases show a high economic burden highlighting need for taking some actions to decrease costs [45–47]. The results of this review showed that CDSSs have the ability to improve laboratory test utilization in some cases including hepatitis B virus, Clostridium Difficile, magnesium, B-Type natriuric peptide, TFT, ESR, and heparin-induced thrombocytopenia tests.

### Laboratory test-related outcomes

Appropriate testing and cost saving were both affected by the CDSSs which is consistent with a similar systematic review on outpatient setting [22]. It is also consistent with a narrative review by Bindraban et al. [48] showing nearly all interventions in educational, CPOE, and audit and feedback category caused reduction in test order volume. Thesystematic review by Roshanov et al. [20] also indicated that those systems aiming at reducing test ordering rate had positive impact. However, the results are inconsistent with Delvaux and colleague systematic review. They found that CDSSs designed to change laboratory testing behavior for diabetes, HIV, and anticoagulation had little or no influence on clinical outcome. Our study included studies aiming at improving laboratory testing process as the primary aim. However, most studies included by Delvaux et al., as mentioned in introduction section, had different objective, for instance computer-aided dosing, and further evaluated its impact on diagnostic testing. Thus, it seems CDSSs specifically designed to affect laboratory tests are more influential. Eaton et al. [36] showed that CDSSs might be effective for some tests and ineffective for some others. There was only one study [42] that found a negative impact in magnesium ordering attributed to CDSS. The CDSS was supposed to regulate magnesium ordering; they developed a CDSS in a way that three tests (i.e. magnesium, calcium, and phosphorus) could be ordered from one user interface of CPOE. This may have caused an unintentional prompt to order these tests together without original plan. Cost reduction in laboratory tests was reported in several studies [28, 30, 32, 34, 37, 38, 41]. But it is important to mention that the quality of the studies was fair and the results were not analyzed statistically. Thus, the conclusion about cost reduction sounds difficult. However, it is stated that the reported cost reduction is an underestimation of true cost savings since they only assessed consumables costs; the associated resources (i.e. equipment, personnel, test tubes, etc.) should be included in the calculation.

### Physician-related outcomes

The studies reporting physician-related outcomes [28, 31, 33] showed positive effect on compliance to the CDSS recommendations. A systematic review by Delvaux et al. [23] also demonstrated a positive impact in compliance with recommendations made by CDSSs. Roshanov et al. [20] also concluded that CDSSs had positive impact on physicians’ diagnostic test ordering behaviors. However, they believed that the contributing factors resulting in success or failure are unclear. Main et al. found that if they consider the result of both primary and secondary outcome then CDSSs is effective on physicians’ behaviors.

### Patient-related outcomes

The results also indicated that the evidence pertaining to the effects of CDSSs on patient-related outcomes is limited. Overall, CDSSs may make little or no difference to patient outcomes including patient complications, patient disposition, or mortality rate [28, 34, 39, 41]. For instance, in the study by Bates et al. [28], three of the eight urinalysis cancelled tests displayed a few red blood cells, while the previous specimen had been negative. It is inferred from these findings that cancelling the orders due to a CDSS suggestion, probably lead to no adverse event to patients. The study by Bridges et al. [34] showed that patients with duplicate tests had higher mortality rate than those without duplicate tests; they also had a worse disposition after discharge, indicating those with redundant tests were generally sicker. Thus, less mortality rate cannot be only attributed to CDSS effect and needs more investigation. Patient experience like decreased phlebotomy and other possible improved outcomes like decreased risk for false-positive test results should be investigated in future studies.

### Strengths and limitations

A comprehensive search strategy, without any time period restriction, was performed to find the maximum number of relevant studies. To avoid missing any important findings, a variety of interventional study designs were included. We assessed the effects of CDSSs not only on proportion of test orders and associated costs but also on physician-related and patient-related clinical outcomes.

A limitation of this review is that due to exclusion of non-English language papers and conference proceedings, some relevant studies might have been missed. Another limitation is the exclusive focus on studies on reducing unnecessary testing as the main outcome. Most studies conducted in this field were performed using a quasi-experimental design making the conclusion about the impacts difficult due to possible biases.

### Implication

Applying a clinical algorithm and hard stop alerts for preventing specified tests would result in more reduction in tests volume. CDSSs should be evaluated for specific laboratory tests to make sure only effective alerts would be displayed [36]. Nonetheless, allowing overrides may be effective for clinicians’ acceptance of the system. Nonintrusive alerts should be evaluated to make sure only effective alerts continue to be displayed so as to prevent rising alert fatigue [36]. Alert fatigue causes both important and non-important alerts to be overridden by clinicians. Thus, considering a balance between system flexibility and hard-stop alerts is important in designing a CDSS. It is suggested that the intervention must be sustainable through providing awareness to the changes, which will bring about better compliance. Impact on physician-related outcomes can be promoted over time, since physicians possibly experience an “adjustment” period at the beginning of the intervention; therefore, they need time to become familiar with the intervention [34]. Although physicians’ attitude and requirements are important factors contributing in more acceptances and perceived usefulness of CDSS, less attention has been paid to them. It has been shown that simple static rules had higher compliance rates than complicated dynamic rules [31]. CDSSs design should not allow two or more tests to be ordered from a single interface, because it may contribute in unintentional prompt to order those tests together and increase tests ordering.

### Future research directions

Since most studies were conducted after 2015, indicating a new research agenda, there is a need for more studies investigating effective information technology-based approaches to manage health resources utilization. Moreover, considering the majority of the studies were performed using a quasi-experimental design, there is an essential need for further studies with more robust study designs. Also, to make sure about the effects of CDSSs on test interval, STAT tests, and TAT, further studies are needed. Considering lack of evidence on potential negative effects resulting from the cancellation of the tests based on CDSS recommendations, future research should evaluate these effects, especially potential harm to patients. Although some physicians need guidance when interpreting some tests [49, 50] and CDSSs have the potential to aid them, according to our review there was no physician aid for interpreting the result; new research can investigate the effects of CDSSs as a physician aid for interpreting the laboratory tests results.

## Conclusion

Current systematic review indicate that CDSSs increase appropriate test ordering through eliminating redundant test orders and enhancing evidence-based practice in hospitals. The literatures showed that CDSSs have the potential to influence on cost savings. However, evidence is limited about the impact of cancelling order tests on patient health and needs further studies. As suggested, there is an essential need for further studies with more robust study designs like randomized controlled trials.

## Supplementary Information

**Additional file 1.** Search strategy.

**Additional file 2.** Quality assessment of the included studies.

## Data Availability

All data are available in the submission.

## Abbreviations

CDSS: Clinical decision support system
EMR: Electronic medical record
EHR: Electronic health record
CPOE: Computerized physician order entry
TAT: Turnaround time
RCT: Randomized controlled trials
CCT: Controlled clinical trials
ITS: Interrupted time series
ED: Emergency department
IG: Intervention group
CG: Control group
TFT: Thyroid function test
TSH: Thyroid stimulation hormone
C. difficile: Clostridium difficile

## References

[1] Zhi M, Ding EL, Theisen-Toupal J, Whelan J, Arnaout R (2013). The landscape of inappropriate laboratory testing: a 15-year meta-analysis. PLoS ONE.

[2] Stilwell JA, Young D, Cunnington A (1980). Evaluation of laboratory tests in hospitals. Ann Clin Biochem.

[3] Nachamkin I. How many lab tests do patients really need? University of Pennsylvania. Pathology and Laboratory Medicine; 2015. http://pathology.med.upenn.edu/department/blogs/pepper-talk/how-many-lab-tests-do-patients-really-need.

[4] Naugler C, Ma I (2018). More than half of abnormal results from laboratory tests ordered by family physicians could be false-positive. Can Fam Phys.

[5] Thavendiranathan P, Bagai A, Ebidia A, Detsky AS, Choudhry NK (2005). Do blood tests cause anemia in hospitalized patients? The effect of diagnostic phlebotomy on hemoglobin and hematocrit levels. J Gen Intern Med.

[6] Kachalia A, Gandhi TK, Puopolo AL, Yoon C, Thomas EJ, Griffey R (2007). Missed and delayed diagnoses in the emergency department: a study of closed malpractice claims from 4 liability insurers. Ann Emerg Med.

[7] Gandhi TK, Kachalia A, Thomas EJ, Puopolo AL, Yoon C, Brennan TA (2006). Missed and delayed diagnoses in the ambulatory setting: a study of closed malpractice claims. Ann Intern Med.

[8] Medicine NAo (2015). Improving diagnosis in health care.

[9] Holland LL, Smith LL, Blick KE (2005). Reducing laboratory turnaround time outliers can reduce emergency department patient length of stay: an 11-hospital study. Am J Clin Pathol.

[10] Meidani Z, Farzandipour M, Hosseinpour M, Kheirkhah D, Shekarchi M, Rafiei S (2017). Evaluating inappropriate patient stay and its reasons based on the appropriateness evaluation protocol. Nurs Midwifery Stud.

[11] Rogg JG, Rubin JT, Hansen P, Liu SW (2013). The frequency and cost of redundant laboratory testing for transferred ED patients. Am J Emerg Med.

[12] Naugler C, Thomas R, Turin TC, Guo M, Vaska M (2015). Yearly clinical laboratory test expenditures for different medical specialties in a major Canadian city. Am J Clin Pathol.

[13] Meidani Z, Farzandipour M, Farrokhian A, Haghighat M (2016). A review on laboratory tests' utilization: a trigger for cutting costs and quality improvement in health care settings. Med J Islam Repub Iran.

[14] Blaya JA, Shin SS, Yagui M, Contreras C, Cegielski P, Yale G (2014). Reducing communication delays and improving quality of care with a tuberculosis laboratory information system in resource poor environments: a cluster randomized controlled trial. PLoS ONE.

[15] Bell DS, Cima L, Seiden DS, Nakazono TT, Alcouloumre MS, Cunningham WE (2012). Effects of laboratory data exchange in the care of patients with HIV. Int J Med Inform.

[16] Turner HE, Deans KA, Kite A, Croal BL (2013). The effect of electronic ordering on pre-analytical errors in primary care. Ann Clin Biochem.

[17] Georgiou A, Lang S, Rosenfeld D, Westbrook JI (2011). The use of computerized provider order entry to improve the effectiveness and efficiency of coagulation testing. Arch Pathol Lab Med.

[18] Hunt D, Haynes R, Hanna S (1998). Effects of computer-based clinical decision support systems on physician performance and patient outcomes. JAMA.

[19] Revolinsk S (2015). Implementation of a clinical decision support alert for the management of Clostridium difficile infection. Antibiotics (Basel).

[20] Roshanov PS, You JJ, Dhaliwal J, Koff D, Mackay JA, Weise-Kelly L (2011). Can computerized clinical decision support systems improve practitioners' diagnostic test ordering behavior? A decision-maker-researcher partnership systematic review. Implement Sci.

[21] Main C, Moxham T, Wyatt JC, Kay J, Anderson R, Stein K (2010). Computerised decision support systems in order communication for diagnostic, screening or monitoring test ordering: systematic reviews of the effects and cost-effectiveness of systems. Health Technol Assess.

[22] Maillet E, Pare G, Currie LM, Raymond L, Ortiz de Guinea A, Trudel MC (2018). Laboratory testing in primary care: a systematic review of health IT impacts. Int J Med Inform.

[23] Delvaux N, Van Thienen K, Heselmans A, de Velde SV, Ramaekers D, Aertgeerts,  (2017). The effects of computerized clinical decision support systems on laboratory test ordering: a systematic review. Arch Path Lab Med.

[24] Payne TH (2000). Computer decision support systems. Chest.

[25] Institute TNHLaB Quality assessment tool. https://www.nhlbi.nih.gov/health-topics/study-quality-assessment-tools.

[26] Goldzweig CL, Orshansky G, Paige NM, Ewing BA, Miake-Lye IM, Beroes JM, et al. VA evidence-based synthesis program reports. Electronic health record-based interventions for reducing inappropriate imaging in the clinical setting: a systematic review of the evidence. Washington: Department of Veterans Affairs (US); 2015. 25973514

[27] Nabovati E, Vakili-Arki H, Taherzadeh Z, Saberi MR, Medlock S, Abu-Hanna A (2017). Information technology-based interventions to improve drug-drug interaction outcomes: a systematic review on features and effects. J Med Syst.

[28] Bates DW, Kuperman GJ, Rittenberg E, Teich JM, Fiskio J, Ma'luf N (1999). A randomized trial of a computer-based intervention to reduce utilization of redundant laboratory tests. Am J Med.

[29] Waldron JL, Ford C, Dobie D, Danks G, Humphrey R, Rolli A (2014). An automated minimum retest interval rejection rule reduces repeat CRP workload and expenditure, and influences clinician-requesting behaviour. J Clin Pathol.

[30] Gottheil S, Khemani E, Copley K, Keeney M, Kinney J, Chin-Yee I, et al. Reducing inappropriate ESR testing with computerized clinical decision support. BMJ Qual Improv Rep. 2016;5(1): u211376–w4582. 10.1136/bmjquality.u211376.w4582 PMC4822023 27096092

[31] Boon-Falleur L, Sokal E, Peters M, Ketelslegers JM. A rule-based decision support application for laboratory investigations management. In: Proceedings symposium on computer applications in medical care; 1995. p. 314–318. PMC2579106 8563292

[32] Lippi G, Brambilla M, Bonelli P, Aloe R, Balestrino A, Nardelli A (2015). Effectiveness of a computerized alert system based on re-testing intervals for limiting the inappropriateness of laboratory test requests. Clin Biochem.

[33] Nies J, Colombet I, Zapletal E, Gillaizeau F, Chevalier P, Durieux P (2010). Effects of automated alerts on unnecessarily repeated serology tests in a cardiovascular surgery department: a time series analysis. BMC Health Serv Res.

[34] Bridges SA, Papa L, Norris AE, Chase SK (2014). Duplicated laboratory tests: evaluation of a computerized alert intervention abstract. J Healthc Qual.

[35] Dalal S, Bhesania S, Silber S, Mehta P. Use of electronic clinical decision support and hard stops to decrease unnecessary thyroid function testing. BMJ Qual Improv Rep. 2017;6(1):u223041.w8346. 10.1136/bmjquality.u223041.w8346 PMC5411717 28469901

[36] Eaton KP, Chida N, Apfel A, Feldman L, Greenbaum A, Tuddenham S (2018). Impact of nonintrusive clinical decision support systems on laboratory test utilization in a large academic centre. J Eval Clin Pract.

[37] Klatte JM, Selvarangan R, Jackson MA, Myers AL (2016). Reducing overutilization of testing for clostridium difficile infection in a pediatric hospital system: a quality improvement initiative. Hosp Pediatr.

[38] Levick DL, Stern G, Meyerhoefer CD, Levick A, Pucklavage D (2013). Reducing unnecessary testing in a CPOE system through implementation of a targeted CDS intervention. BMC Med Inform Decis Mak.

[39] Nicholson MR, Freswick PN, Di Pentima MC, Wang L, Edwards KM, Wilson GJ (2017). The use of a computerized provider order entry alert to decrease rates of clostridium difficile testing in young pediatric patients. Infect Control Hosp Epidemiol.

[40] Quan KA, Yim J, Merrill D, Khusbu U, Madey K, Dickey L (2018). Reductions in clostridium difficile infection (CDI) rates using real-time automated clinical criteria verification to enforce appropriate testing. Infect Control Hosp Epidemiol.

[41] Procop GW, Yerian LM, Wyllie R, Harrison AM, Kottke-Marchant K (2014). Duplicate laboratory test reduction using a clinical decision support tool. Am J Clin Pathol.

[42] Rosenbloom ST, Chiu KW, Byrne DW, Talbert DA, Neilson EG, Miller RA (2015). Interventions to regulate ordering of serum magnesium levels: report of an unintended consequence of decision support. JAMIA.

[43] Rudolf JW, Dighe AS, Coley CM, Kamis IK, Wertheim BM, Wright DE (2017). Analysis of daily laboratory orders at a large urban academic center: a multifaceted approach to changing test ordering patterns. Am J Clin Pathol.

[44] Samuelson BT, Glynn E, Holmes M, White AA, Martin DB, Garcia D (2015). Use of a computer-based provider order entry (CPOE) intervention to optimize laboratory testing in patients with suspected heparin-induced thrombocytopenia. Thromb Res.

[45] Murata K, Hinotsu S, Sadamasa N, Yoshida K, Yamagata S, Asari S (2017). Healthcare resource utilization and clinical outcomes associated with acute care and inpatient rehabilitation of stroke patients in Japan. Int J Qual Health Care.

[46] Nguyen MH, Burak Ozbay A, Liou I, Meyer N, Gordon SC, Dusheiko G (2019). Healthcare resource utilization and costs by disease severity in an insured national sample of US patients with chronic hepatitis B. J Hepatol.

[47] Chen K, Krasner A, Li N, Xiang CQ, Totev T, Xie J (2019). Clinical burden and healthcare resource utilization among patients with chronic hypoparathyroidism, overall and by adequately vs not adequately controlled disease: a multi-country chart review. J Med Econ.

[48] Bindraban R, Berg M, Naaktgeboren C (2018). Reducing test utilization in hospital settings: a narrative review. Ann Lab Med.

[49] McGlynn EA, Asch SM, Adams J, Keesey J, Hicks J, DeCristofaro A (2003). The quality of health care delivered to adults in the United States. N Engl J Med.

[50] Schmidt RL, Garcia CA, Panlener J, Ashwood ER, Jackson BR, Hussong JW (2014). An analysis of clinical consultation activities in clinical chemistry: implications for transformation and resident training in chemical pathology. Arch Pathol Lab Med.

