# Supplementary material for: Laboratory test ordering in inpatient hospitals: a systematic review on the effects and features of clinical decision support systems
Source: BMC Med Inform Decis Mak. 2021 Jan 18;21:20. doi: 10.1186/s12911-020-01384-8 (PMC7814592; doi:10.1186/s12911-020-01384-8)
Supplement: Supplementary file 1 — Additional file 1. Search strategy. [file 12911_2020_1384_MOESM1_ESM.docx]

**Supplementary A: Search strategy**

Data base: Medline through PubMed

Date: 2020/01/21

Filter: English

Result: 1036

Sort: Most recent

Imported: 1036

Search query:

(Clinical Laboratory Techniques[Mesh] OR Laboratories[Mesh] OR "Clinical Laboratory Services"[Mesh] OR "Diagnostic Tests, Routine"[Mesh] OR Laborator*[TITLE/ABSTRACT] OR "Diagnostic Test"[TITLE/ABSTRACT] OR "Diagnostic Tests"[TITLE/ABSTRACT] OR "diagnostic Testing"[TITLE/ABSTRACT] OR "Diagnosis Test"[TITLE/ABSTRACT] OR "Diagnosis Tests"[TITLE/ABSTRACT] OR "diagnosis Testing"[TITLE/ABSTRACT] OR "Test order" [TITLE/ABSTRACT] OR "Test-ordering"[TITLE/ABSTRACT] OR "Test ordering"[TITLE/ABSTRACT]) AND (clinical decision support systems[MESH] OR "decision support system"[TITLE/ABSTRACT] OR "decision support tool"[TITLE/ABSTRACT] OR "reminder system"[TITLE/ABSTRACT] OR "reminding system"[TITLE/ABSTRACT] OR "alert system"[title/abstract] OR "alerting system"[title/abstract] OR computer assisted decision making[MESH] OR "assisted decision making"[TITLE/ABSTRACT] OR diagnosis, computer assisted[MESH] OR expert systems[MESH] OR "expert system"[TITLE/ABSTRACT] OR CDS*[TITLE/ABSTRACT] OR medical order entry systems[MESH] OR "order entry system"[TITLE/ABSTRACT] OR "computerized order entry"[TITLE/ABSTRACT] OR "computerized prescriber order entry"[TITLE/ABSTRACT] OR "computerized provider order entry"[TITLE/ABSTRACT] OR "computerized physician order entry"[TITLE/ABSTRACT] OR "electronic order entry"[TITLE/ABSTRACT] OR "automated order entry"[TITLE/ABSTRACT] OR CPOE[TITLE/ABSTRACT] OR electronic prescribing[MESH] OR "electronic prescribing"[TITLE/ABSTRACT] OR "electronic prescription"[TITLE/ABSTRACT] OR "computer assisted therapy"[TITLE/ABSTRACT] OR "computer assisted diagnosis"[TITLE/ABSTRACT]) AND (Hospitals[Mesh] OR Inpatients[Mesh] OR Hospital*[ TITLE/ABSTRACT] OR Inpatient*[TITLE/ABSTRACT])

Database: Scopus

Date: 2020/01/21

Filter: English, Source type: journal, Document type: article

Result: 1357

Imported after Pubmed: 921

Search query:

TITLE-ABS-KEY ((Laborator* OR "Diagnostic Test" OR "Diagnostic Tests" OR "diagnostic Testing" OR "Diagnosis Test" OR "Diagnosis Tests" OR "diagnosis Testing" OR "Test order" OR "Test-ordering" OR "Test ordering") AND ("decision support system" OR "decision support tool" OR "reminder system" OR "reminding system" OR "alert system" OR "alerting system" OR "expert system" OR CDS* OR "order entry system" OR "computerized order entry" OR "computerized prescriber order entry" OR "computerized provider order entry" OR "computerized physician order entry" OR "electronic order entry" OR "automated order entry" OR CPOE OR "electronic prescribing" OR "electronic prescription" OR "computer assisted therapy" OR "computer assisted drug" OR "computer assisted diagnosis") AND (Hospital* OR Inpatient*))

Database: ISI

Date: 2020/01/21

Filter: English, Document type: article

Result: 279

Imported: 74 after Pubmed and Scopus

Search query:

(Laborator* OR "Diagnostic Test" OR "Diagnostic Tests" OR "diagnostic Testing" OR "Diagnosis Test" OR "Diagnosis Tests" OR "diagnosis Testing" OR "Test order" OR "Test-ordering" OR "Test ordering") AND ("decision support system" OR "decision support tool" OR "reminder system" OR "reminding system" OR "alert system" OR "alerting system" OR "expert system" OR CDS* OR "order entry system" OR "computerized order entry" OR "computerized prescriber order entry" OR "computerized provider order entry" OR "computerized physician order entry" OR "electronic order entry" OR "automated order entry" OR CPOE OR "electronic prescribing" OR "electronic prescription" OR "computer assisted therapy" OR "computer assisted drug" OR "computer assisted diagnosis") AND (Hospital* OR Inpatient*)

Database: Cochrane

Date: 2020/01/21

Filter: None

Result: 76

Imported: 42 after Pubmed, Scopus and ISI

Search query:

(Laborator* OR "Diagnostic Test" OR "Diagnostic Tests" OR "diagnostic Testing" OR "Diagnosis Test" OR "Diagnosis Tests" OR "diagnosis Testing" OR "Test order" OR "Test-ordering" OR "Test ordering") AND ("decision support system" OR "decision support tool" OR "reminder system" OR "reminding system" OR "alert system" OR "alerting system" OR "expert system" OR CDS* OR "order entry system" OR "computerized order entry" OR "computerized prescriber order entry" OR "computerized provider order entry" OR "computerized physician order entry" OR "electronic order entry" OR "automated order entry" OR CPOE OR "electronic prescribing" OR "electronic prescription" OR "computer assisted therapy" OR "computer assisted drug" OR "computer assisted diagnosis") AND (Hospital* OR Inpatient*)
