# Supplementary material for: Laboratory test ordering in inpatient hospitals: a systematic review on the effects and features of clinical decision support systems
Source: BMC Med Inform Decis Mak. 2021 Jan 18;21:20. doi: 10.1186/s12911-020-01384-8 (PMC7814592; doi:10.1186/s12911-020-01384-8)
Supplement: Supplementary file 2 — Additional file 2. Quality assessment of the included studies. [file 12911_2020_1384_MOESM2_ESM.docx]

| criteria  **Supplementary B: Quality assessment of the included studies*** | Bridge  (34) | Boon  (31) | Dalal  (35) | Eaton  (36) | Gottheil  (30) | Klatte  (37) | Levick  (38) | Lippi  (32) | Nies  (33) | Quan  (40) | Procop  (41) | Rosenbloom  (42) | Rudolf  (43) | Samuelson  (44) |
| --- | --- | --- | --- | --- | --- | --- | --- | --- | --- | --- | --- | --- | --- | --- |
| 1. Was the study question or objective clearly stated? | yes | yes | yes | yes | yes | yes | yes | yes | yes | yes | yes | yes | yes | yes |
| 2. Were eligibility/selection criteria for the study population prespecified and clearly described? | yes | yes | yes | yes | no | yes | no | no | no | no | no | yes | no | yes |
| 3. Were the participants in the study representative of those who would be eligible for the test/service/intervention in the general or clinical population of interest? | yes | yes | yes | yes | yes | yes | yes | yes | yes | yes | yes | yes | yes | yes |
| 4. Were all eligible participants that met the prespecified entry criteria enrolled? | yes | yes | yes | yes | yes | yes | yes | yes | yes | yes | yes | yes | yes | yes |
| 5. Was the sample size sufficiently large to provide confidence in the findings? | yes | no | yes | NR | NR | NR | NR | NR | no | NR | NR | NR | NR | NR |
| 6. Was the test/service/intervention clearly described and delivered consistently across the study population? | yes | yes | yes | yes | yes | yes | yes | yes | yes | yes | yes | yes | yes | yes |
| 7. Were the outcome measures **prespecified,** clearly defined, valid, reliable, and assessed consistently across all study participants? | yes | yes | yes | yes | yes | yes | yes | yes | yes | yes | yes | yes | yes | yes |
| 8. Were the people assessing the outcomes blinded to the participants' exposures/interventions? | no | no | no | no | no | no | no | no | no | no | no | no | no | no |
| 9. Was the loss to follow-up after baseline 20% or less? Were those lost to follow-up accounted for in the analysis? | yes | yes | yes | yes | yes | yes | yes | yes | yes | yes | yes | yes | yes | yes |
| 10. Did the statistical methods examine changes in outcome measures from before to after the intervention? Were statistical tests done that provided p values for the pre-to-post changes? | yes | no | yes | yes | no | no | yes | yes | yes | yes | yes | yes | yes | yes |
| 11. Were outcome measures of interest taken multiple times before the intervention and multiple times after the intervention (i.e., did they use an interrupted time-series design)? | yes | no | no | yes | yes | yes | yes | no | yes | yes | yes | yes | yes | no |
| 12. If the intervention was conducted at a group level (e.g., a whole hospital, a community, etc.) did the statistical analysis take into account the use of individual-level data to determine effects at the group level? | NA | NA | NA | NA | NA | NA | NA | NA | NA | NA | NA | NA | NA | NA |
| TOTAL | Good | Fair | Fair | good | Fair | Fair | Fair | Fair | Fair | Fair | Fair | good | Fair | Fair |

* The RCT (27) and case-control (38) studies were assessed by different tools so, only the quality assessments of the time series and before-after studies with no control group are presented in this table. Both studies were evaluated as good quality.
